# Genome sequences of 70 multidrug-resistant Gram-negative isolates in high-risk neonates in the Northeast of Mexico

**DOI:** 10.1128/mra.00274-24

**Published:** 2024-09-03

**Authors:** Lorena Rodriguez-Orduña, Victor Javier Lara-Diaz, Mario René Alcorta-Garcia, Claudia Nohemi Lopez-Villaseñor, Cuauhtemoc Licona-Cassani

**Affiliations:** 1 Centro de Biotecnología FEMSA, Escuela de Ingeniería y Ciencias, Tecnologico de Monterrey, Nuevo León, Mexico; 2 Faculty of Medicine, Discipline of Paediatrics and Child Health, University of New South Wales, Sydney, Australia; 3 Escuela de Medicina y Ciencias de la Salud, Tecnologico de Monterrey, Monterrey, Nuevo León, Mexico; 4 Hospital Regional Materno Infantil, Servicios de Salud de Nuevo León, Guadalupe, Nuevo León, Mexico; University of Maryland School of Medicine, Baltimore, Maryland, USA

**Keywords:** drug resistance, microbial, infant, newborn, Gram-negative bacteria, perinatal care

## Abstract

Infections by multidrug-resistant pathogens are steadily increasing worldwide. A considerable proportion of neonatal intensive care admissions have a bacterial infection with multidrug-resistant bacteria during their hospital stay. In this work, we report draft genome sequences of 70 selected isolates from high-risk neonates in the Northeast of Mexico.

## ANNOUNCEMENT

The increasing prevalence of multidrug-resistant (MDR) pathogens is a global concern (1), particularly in resource-limited hospitals (2, 3). Bacteremia caused by MDR Gram-negatives correlates with high mortality (4). In Mexico, a high prevalence of resistant Gram-negative bacteria in neonatal intensive care units (NICU) has been reported (5). In 2020, our group observed an increase in MDR Gram-negative isolates among hospitalized neonates in a third-level hospital in Mexico. Socio-economic factors contribute to inadequate prenatal care, leading to NICU admissions exceeding 9% of live births. Empirical antibiotic use raises the risk of bacterial infection and antimicrobial resistance. To establish resistance prevalence, we conducted a prospective audit investigating antimicrobial resistance in Gram-negative clinical isolates at a Mexican hospital.

Strains were isolated from neonates under critical care at the Hospital Regional Materno Infantil in Nuevo León, Mexico (25.69 N 100.22 W), from 2021 to 2022. Blood samples drawn in BD Bactec-Peds Plus/F culture vials were kept in the Bactec FX40 system until isolation or up to 7 days; positive samples were inoculated into blood agar, chocolate agar, Mac Conkey, S-110, and Potato Dextrose Agar (PDA) agar plates and incubated at 37°C ± 1.5°C and aerobic conditions for up to 72 hours. Non-blood samples were inoculated into Mac Conkey, S.S., brilliant green, PDA, bismuth sulfite agar, tetrathionate broth, and thioglycolate broth and incubated as stated above (6). Isolated microorganisms were transferred to the VITEK2 compact system, for identification and antibiotic susceptibility tests, and validated by the Advanced expert system. Each isolate was cryo-preserved at −80°C in glycerol-based media until DNA extraction. Thawed samples were plated on Luria Bertani (LB) agar and incubated at 37°C for 24 hours; then, a colony was inoculated on liquid media LB and grown at 37°C for 20 hours. Biomass was collected, and genomic DNA was extracted using the PureLink Genomic DNA Mini Kit (Thermo Fisher Scientific, K182001) following the manufacturer’s instructions. DNA quantification was performed using Qubit v.3 (Invitrogen, California, USA), and integrity was confirmed by gel electrophoresis. Libraries were prepared utilizing the Illumina DNA Prep kit and IDT 10 bp UDI indices and sequenced on Illumina NextSeq 2000, producing 2 × 151-bp reads.

The reads were processed in Trimmomatic v0.33 (7) to remove sequences with QC less than 20 and adapter sequences. Assembly was conducted in the BV-BRC server (https://www.bv-brc.org/) (8) utilizing Unicycler v0.4.8 (9) and Pilon v1.23 (10). Assembly quality was evaluated with Quast v5.2.0 (11) retaining contigs exceeding 300 bp. Genome annotation was performed in PGAP software v6.6 (12). The ANI value and best match type strain were determined during the genome submission process to GenBank. Incoming genomes were compared to existing type assemblies available in RefSeq (13) (February 2024) using a fast MinHash-based k-mer comparison (14). Subsequently, MegaBLAST (15) alignment was performed to obtain the percent identity. BLAST 2.15.0 was used with default parameters. Our data set had an average genome coverage of 139.12×, an average of 104.05 contigs, and a N50 value of 319,823. Detailed statistical metrics for each genome sequence are in Table 1. Default parameters were used for all software unless otherwise specified.

**TABLE 1:** Statistical genomic information of the 76 Gram-negative strains

Sample name	SRA accession	Assembly accession numbers	Best match organism (NCBI taxonomy)	% ANI	Number of reads	Genome total length (bp)	Genome coverage	Contigs	N50 value	GC content (%)	Antibiotic group resistance^*a*^
Escherichia_coli_GN-003	SRR28299581	GCA_036882565.1	*Escherichia coli KTE26*	98.31	1,563,606,256	5,313,742	294.26	202	147,648	50.53	1, 2
Klebsiella_pneumoniae_GN-004	SRR28299561	GCA_036882525.1	*Klebsiella pneumoniae* subsp. *pneumoniae*	99.16	376,301,748	5,535,109	67.98	96	327,308	57.15	1, 2, 4, 5
Pseudomonas_aeruginosa_GN-011	SRR28299545	GCA_036882545.1	*Pseudomonas aeruginosa*	98.94	1,018,545,107	6,769,106	150.47	112	319,826	66.07	1, 2, 3, 4
Klebsiella_pneumoniae_GN-012	SRR28299559	GCA_036882505.1	*Klebsiella pneumoniae* subsp*. pneumoniae*	99.15	688,921,241	5,597,441	123.08	68	273,305	57.17	1, 2, 5
Klebsiella_pneumoniae_GN-013	SRR28299558	GCA_036882485.1	*Klebsiella pneumoniae* subsp*. pneumoniae*	99.25	1,434,960,929	5,348,363	268.30	124	194,773	57.19	1, 2
Serratia_marcescens_GN-014	SRR28299602	GCA_036882465.1	*Serratia marcescens*	95.56	630,113,135	5,075,663	124.14	28	413,548	60.12	1, 2
Klebsiella_pneumoniae_GN-015	SRR28299557	GCA_036882425.1	*Klebsiella pneumoniae* subsp*. pneumoniae*	99.18	1,445,618,029	5,553,933	260.29	138	213,260	57.18	1
Klebsiella_pneumoniae_GN-016	SRR28299556	GCA_036882405.1	*Klebsiella pneumoniae* subsp*. pneumoniae*	99.17	1,642,709,279	5,508,466	298.22	118	253,179	57.27	1, 2
Klebsiella_pneumoniae_GN-017	SRR28299555	GCA_036882445.1	*Klebsiella pneumoniae* subsp*. pneumoniae*	99.16	2,789,465,945	5,538,905	503.61	92	383,629	57.14	1, 2, 4, 5
Klebsiella_pneumoniae_GN-019	SRR28299554	GCA_036882385.1	*Klebsiella pneumoniae* subsp*. pneumoniae*	99.22	1,184,685,092	5,447,192	217.49	70	355,652	57.29	1, 2, 4, 5
Klebsiella_pneumoniae_GN-020	SRR28299553	GCA_036882325.1	*Klebsiella pneumoniae* subsp*. pneumoniae*	99.22	447,437,616	5,446,553	82.15	73	355,652	57.29	1, 2, 4, 5
Klebsiella_pneumoniae_GN-021	SRR28299552	GCA_036882305.1	*Klebsiella pneumoniae* subsp*. pneumoniae*	99.22	604,996,363	5,446,784	111.07	73	355,652	57.29	1, 2, 4, 5
Klebsiella_pneumoniae_GN-022	SRR28299551	GCA_036882345.1	*Klebsiella pneumoniae* subsp*. pneumoniae*	99.21	975,348,530	5,461,770	178.58	76	369,264	57.2	1, 2, 5
Escherichia_coli_GN-028	SRR28299580	GCA_036882285.1	*Escherichia coli ETEC H10407*	99.39	1,635,162,920	4,951,310	330.25	102	203,943	50.51	1, 2, 4
Escherichia_coli_GN-029	SRR28299579	GCA_036882365.1	*Escherichia coli DSM 30083*	99.86	1,313,274,099	5,332,430	246.28	189	190,550	50.7	1, 2, 4, 5
Enterobacter_cloacae_GN-035	SRR28299601	GCA_036882265.1	*Enterobacter asburiae*	97.03	1,344,853,341	5,193,970	258.93	123	198,079	55.21	1, 2, 6
Escherichia_coli_GN-045	SRR28299578	GCA_036882225.1	*Escherichia coli DSM 30083*	99.84	498,639,156	5,179,188	96.28	105	225,781	50.64	1, 2, 4, 5
Enterobacter_cloacae_GN-049	SRR28299593	GCA_036882145.1	*Enterobacter asburiae*	97.03	460,149,389	5,275,741	87.22	128	187,343	55.16	1, 2, 4, 6
Enterobacter_hormaechei_GN-050	SRR28299608	GCA_036882105.1	*Enterobacter hormaechei* subsp*. steigerwaltii*	99.11	537,973,582	4,766,290	112.87	27	677,887	55.6	1, 2, 3
Enterobacter_hormaechei_GN-053	SRR28299592	GCA_036882125.1	*Enterobacter hormaechei* subsp*. xiangfangensis*	99.07	671,479,277	4,940,953	135.90	93	211,123	55.24	1, 2, 6
Escherichia_coli_GN-054	SRR28299576	GCA_036882085.1	*Escherichia coli DSM 30083*	99.89	507,412,254	5,415,616	93.69	180	194,633	50.48	1, 2, 6
Enterobacter_hormaechei_GN-056	SRR28299590	GCA_036882045.1	*Enterobacter hormaechei* subsp*. steigerwaltii*	98.97	508,166,670	4,926,325	103.15	103	223,512	55.28	1, 2
Escherichia_coli_GN-066	SRR28299574	GCA_036882025.1	*Escherichia coli KTE26*	99.74	411,593,512	5,273,971	78.04	212	136,606	50.77	1
Pseudomonas_aeruginosa_GN-067	SRR28299544	GCA_036880245.1	*Pseudomonas aeruginosa*	99.42	1,259,055,615	6,351,005	198.25	63	311,848	66.45	1
Serratia_marcescens_GN-069	SRR28299600	GCA_036880275.1	*Serratia marcescens* subsp*. marcescens ATCC 13880*	95.58	405,209,003	5,202,593	77.89	28	494,393	59.88	1, 2
Pseudomonas_aeruginosa_GN-071	SRR28299543	GCA_036880255.1	*Pseudomonas aeruginosa*	99.42	1,079,602,798	6,363,915	169.64	43	429,872	66.46	1, 2
Enterobacter_hormaechei_GN-072	SRR28299589	GCA_036880215.1	*Enterobacter hormaechei* subsp*. steigerwaltii*	99.04	518,256,199	4,819,790	107.53	46	464,040	55.4	1, 2, 3, 6
Enterobacter_hormaechei_GN-073	SRR28299588	GCA_036880205.1	*Enterobacter hormaechei* subsp*. hoffmannii*	99.17	515,916,870	4,951,197	104.20	103	148,522	55.02	1, 2
Klebsiella_michiganensis_GN-076	SRR28299563	GCA_036880185.1	*Klebsiella michiganensis*	98.74	561,465,879	6,506,908	86.29	170	148,883	55.42	1, 4, 5
Pseudomonas_aeruginosa_GN-077	SRR28299542	GCA_036880145.1	*Pseudomonas aeruginosa*	99.42	1,047,340,882	6,363,649	164.58	45	444,753	66.46	1, 2
Klebsiella_michiganensis_GN-078	SRR28299562	GCA_036880125.1	*Klebsiella michiganensis*	98.74	463,646,984	6,511,969	71.20	167	157,876	55.42	1, 4
Escherichia_coli_GN-079	SRR28299573	GCA_036880165.1	*Escherichia coli KTE26*	99.74	458,942,402	5,342,175	85.91	215	210,458	50.63	1, 4
Enterobacter_cloacae_GN-083	SRR28299591	GCA_036880105.1	*Enterobacter asburiae*	97.03	531,689,068	5,195,480	102.34	126	198,079	55.21	1, 2
Enterobacter_roggenkampii_GN-084	SRR28299583	GCA_036880005.1	*Enterobacter roggenkampii*	98.62	400,381,729	5,041,596	79.42	142	95,515	55.56	1, 2
Escherichia_coli_GN-085	SRR28299572	GCA_036880015.1	*Escherichia coli DSM 30083*	99.84	414,781,292	5,176,487	80.13	127	211,018	50.64	1
Enterobacter_hormaechei_GN-086	SRR28299587	GCA_036880035.1	*Enterobacter hormaechei* subsp*. steigerwaltii*	99.04	468,706,643	5,114,811	91.64	113	285,709	54.84	1, 2
Escherichia_coli_GN-089	SRR28299570	GCA_036880045.1	*Escherichia coli KTE26*	99.66	428,105,088	5,339,923	80.17	181	192,694	50.58	1
Enterobacter_hormaechei_GN-091	SRR28299586	GCA_036880025.1	*Enterobacter hormaechei* subsp*. steigerwaltii*	99.28	431,700,032	4,812,705	89.70	55	306,885	55.2	1, 2
Pseudomonas_aeruginosa_GN-092	SRR28299541	GCA_036879985.1	*Pseudomonas aeruginosa*	99.41	941,334,032	6,427,381	146.46	97	248,941	66.31	1, 2
Pseudomonas_aeruginosa_GN-093	SRR28299540	GCA_036879925.1	*Pseudomonas aeruginosa*	99.45	1,420,118,965	6,256,975	226.97	79	272,242	66.54	1, 2
Pseudomonas_aeruginosa_GN-094	SRR28299539	GCA_036879915.1	*Pseudomonas aeruginosa*	99.45	1,395,506,660	6,262,105	222.85	77	325,537	66.53	1, 2
Escherichia_coli_GN-095	SRR28299569	GCA_036879905.1	*Escherichia coli DSM 30083*	99.89	374,130,711	5,450,009	68.65	187	190,814	50.5	1, 2, 5
Acinetobacter_baumannii_GN-102	SRR28299610	GCA_036879965.1	*Acinetobacter baumannii ATCC 19606*	97.97	366,023,217	3,656,056	100.11	190	48,867	38.96	1, 4
Pseudomonas_aeruginosa_GN-104	SRR28299537	GCA_036879845.1	*Pseudomonas aeruginosa*	99.45	1,867,302,080	6,256,250	298.47	74	371,942	66.53	1, 2
Serratia_bockelmannii_GN-108	SRR28299603	GCA_036879885.1	*Serratia bockelmannii*	98.86	430,328,082	5,374,157	80.07	34	425,434	59.13	1, 2
Escherichia_coli_GN-110	SRR28299568	GCA_036879805.1	*Escherichia coli KTE26*	99.74	393,442,964	5,211,410	75.50	141	165,266	50.61	1, 4, 5
Acinetobacter_baumannii_GN-114	SRR28299609	GCA_036879865.1	*Acinetobacter baumannii*	98.62	385,539,192	3,818,850	100.96	154	54,674	38.84	1
Citrobacter_amalonaticus_GN-115	SRR28299571	GCA_036879775.1	*Citrobacter amalonaticus*	99.16	413,274,944	5,386,960	76.72	126	253,891	53.01	1, 2, 4
Citrobacter_portucalensis_GN-116	SRR28299560	GCA_036879765.1	*Citrobacter portucalensis*	98.58	617,425,728	5,127,720	120.41	95	111,320	52.07	1
Escherichia_coli_GN-118	SRR28299566	GCA_036879705.1	*Escherichia coli KTE26*	99.68	520,475,372	5,453,933	95.43	222	242,570	50.61	1
Serratia_ureilytica_GN-122	SRR28299598	GCA_036879715.1	*Serratia ureilytica*	99.03	408,348,051	5,141,834	79.42	23	1,410,298	59.58	1
Klebsiella_pneumoniae_GN-128	SRR28299550	GCA_036879745.1	*Klebsiella pneumoniae* subsp*. pneumoniae*	99.14	441,017,498	5,617,792	78.50	104	222,408	56.91	1, 2, 5
Klebsiella_pneumoniae_GN-131	SRR28299548	GCA_036879685.1	*Klebsiella pneumoniae* subsp*. pneumoniae*	99.21	456,271,137	5,215,706	87.48	85	173,023	57.48	1, 6
Serratia_ureilytica_GN-135	SRR28299597	GCA_036879655.1	*Serratia ureilytica*	99.04	473,343,041	5,099,548	92.82	22	730,194	59.69	1, 2
Escherichia_coli_GN-137	SRR28299564	GCA_036879605.1	*Escherichia coli KTE26*	99.74	516,301,640	5,032,561	102.59	125	210,929	50.6	1, 2
Klebsiella_pneumoniae_GN-138	SRR28299547	GCA_036879625.1	*Klebsiella pneumoniae* subsp*. pneumoniae*	99.2	384,174,508	5,457,280	70.40	53	357,557	57.19	1
Serratia_ureilytica_GN-139	SRR28299596	GCA_036879515.1	*Serratia ureilytica*	99.02	285,430,247	5,129,725	55.64	24	2,930,616	59.6	1, 2
Serratia_nematodiphila_GN-140	SRR28299599	GCA_036879535.1	*Serratia nematodiphila DZ0503SBS1*	97.92	557,856,879	5,064,320	110.15	39	511,575	59.8	1, 2
Klebsiella_pneumoniae_GN-142	SRR28299546	GCA_036879525.1	*Klebsiella pneumoniae* subsp*. pneumoniae*	99.16	493,262,096	5,548,923	88.89	154	252,773	57.12	1, 6
Pseudomonas_aeruginosa_GN-150	SRR28299536	GCA_036879505.1	*Pseudomonas aeruginosa*	99.37	1,019,496,767	6,946,137	146.77	98	23,398	65.99	1, 2, 4, 5
Pseudomonas_aeruginosa_GN-155	SRR28299535	GCA_036879545.1	*Pseudomonas aeruginosa*	99.37	1,536,280,250	6,940,526	221.35	98	217,631	65.99	1, 2
Enterobacter_hormaechei_GN-156	SRR28299585	GCA_036879435.1	*Enterobacter hormaechei* subsp*. oharae*	98.49	319,518,479	4,635,429	68.93	67	192,352	55.55	1, 2
Pseudomonas_aeruginosa_GN-158	SRR28299534	GCA_036879445.1	*Pseudomonas aeruginosa*	99.37	471,068,508	6,951,888	67.76	95	240,917	65.98	1, 2
Enterobacter_asburiae_GN-159	SRR28299549	GCA_036879425.1	*Enterobacter asburiae*	97.07	1,011,580,232	5,074,382	199.35	152	130,428	55.19	1, 2, 6
Pseudomonas_aeruginosa_GN-162	SRR28299607	GCA_036879415.1	*Pseudomonas aeruginosa*	99.5	1,240,671,742	7,155,157	173.40	140	181,796	65.68	1, 2, 3, 4
Pseudomonas_guariconensis_GN-165	SRR28299604	GCA_036879405.1	*Pseudomonas guariconensis*	98.08	1,162,991,376	5,119,372	227.17	28	330,935	62.54	1, 2, 6
Enterobacter_hormaechei_GN-173	SRR28299584	GCA_036879295.1	*Enterobacter hormaechei* subsp*. oharae*	98.51	466,380,418	4,867,474	95.82	53	305,766	55.28	1, 2, 4, 5
Enterobacter_asburiae_GN-174	SRR28299538	GCA_036879275.1	*Enterobacter asburiae*	97.07	445,165,040	4,756,229	93.60	118	144,711	55.70	1, 2
Pseudomonas_aeruginosa_GN-178	SRR28299606	GCA_036879305.1	*Pseudomonas aeruginosa*	99.48	833,388,193	6,421,129	129.79	83	312,035	66.4	1, 2, 6
Pseudomonas_aeruginosa_GN-214	SRR28299605	GCA_036879265.1	*Pseudomonas aeruginosa*	99.39	835,287,480	6,664,325	125.34	91	284,102	66.2	1, 2

^*a*^ 1, penicillins; 2, cephalosporines; 3, monobactams/carbapenems; 4, aminoglycosides; 5, quinolones; 6, fosfomycin.

## Data Availability

This whole genome shotgun project has been deposited in NCBI under BioProject PRJNA1076266. Additionally, the Biosample Accession and SRA Accession numbers for the raw reads are listed in Table 1. The version described in this paper is the first version.
